# Supplementary material for: Patient-reported factors associated with avoidance of in-person care during the COVID-19 pandemic: Results from a national survey
Source: PLoS One. 2022 Aug 5;17(8):e0272609. doi: 10.1371/journal.pone.0272609 (PMC9355185; doi:10.1371/journal.pone.0272609)
Supplement: Attachment — Submitted filename: Response to reviewer comments.docx [file pone.0272609.s002.docx]

Our thanks to the reviewers for the excellent and constructive comments on our manuscript. Below please find a point-by-point response to each comment. Please note that line references refer to the Revised Manuscript with Track Changes document.

**Reviewer #1 comments:**
Overall an interesting piece of work that provides insights into patient perspective on in-person care avoidance during a pandemic. The results can potentially feed into care provider policies and improve services by addressing patient concerns and apprehensions during the current pandemic and beyond.
1. Methodology: It is not entirely clear whether telemedicine is being considered a sub-set of in-person care for the purpose of analysis in this study. Line # 337-338. For example, in primary care, a large majority of in-person care visits were transferred to telemedicine visits. Further, as in reference #12, (Alhadramy OM, Sr. The Structure and the Outcome of Telephone-Based Cardiac Consultations During Lockdown: A Lesson From COVID-19. Cureus. 2020), the conclusion states that: When standard face-to-face cardiac consultations are compromised, a structured telephone-based consultation is considered feasible, seems effective, and promising alternative method of delivering the utmost cardiac care to the community. It might be helpful if the authors mention that whether telemedicine was considered suitable alternative to physical in-person care, and how this has been dealt in the analysis.

*Response: This study focused exclusively on avoidance of in-person visits among those patients who reported needing in-person care. These did not include telemedicine visits. We now reiterate this in the Methods where we define the outcome variable, line 109-110. We have also added a sentence at the end of the Limitations section, lines 401-403, stating that while telehealth visits have been deemed feasible for some patients (citing Alhadramy, now reference #34), our study focused exclusively on avoidance of in-person visits.*
2. Discussions:
a. It might be good to draw comparisons with results from other similar study at reference #7 (Czeisler ME, Marynak K, Clarke KEN, Salah Z, Shakya I, Thierry JM, et al. Delay or Avoidance of Medical Care Because of COVID-19-Related Concerns - United States, June 2020. MMWR Morb Mortal Wkly Rep. 2020). This would specifically be regarding
i. Is the current discussion on avoidance of in-person care, a subset of care avoidance discussed in reference #7?
*Response: Thank you for this excellent comment. In the Introduction, lines 44-54, we have included more detail regarding the previous studies by Czeisler et al (reference #7) and Ganson et al (reference #19), both of which conducted national surveys on delay and/or avoidance of medical care during the pandemic. In the second paragraph of the Discussion, we now compare our results to those of Czeisler et al and Gannon et al, suggesting possible reasons for the differences.*

ii. What is the possible influence of use of different tools for data collection on the results?
*Response: This is now discussed in the second paragraph of the Discussion section where we compare our results to those of others who conducted national surveys.*

b. Line# 291-293: Our hypothesized model also included environment-related variables such as consuming COVID-related media news and the politics related to the pandemic, as we reasoned they might instill fear and panic, thereby influencing care avoidance. Although, as per the analysis, the variable did not have influence on patient decision in the analysis, this could have contributed to COVID related stress that had influence.
*Response: Thank you for this comment. The media and politics variables were, indeed, significantly correlated with the COVID-related stress scores (p<.001). However, as mentioned in the Results, consuming COVID-related content on news media (line 259) was excluded from the regression analysis due to lack of an independent relationship with care avoidance; exposure to COVID-related politics was excluded due to multicollinearity (line 269). We now mention in the Discussion (lines 342-345) that the stress score, comprised of variables that centered on physical risks of contagion, could have been influenced by those variables.*

c. Kindly clarify if institutional factors like prioritization for COVID-19 patients and resource diversion could have been a factor for avoidance of in-person care?
*Response: Thank you for this interesting comment.* *We did not specifically ask about this in our survey, focusing instead on participant exposure to media campaigns from local healthcare organizations ensuring patients of the safety protocols that were in place. We now mention this in the Discussion section, lines 370-381, mentioning three new references that support the possibility of resource diversion at certain times during the pandemic.*

d. Out of the seven key factors found to be associated with care avoidance, age, ability to afford care and HCO experience are not directly COVID-19 related. However, COVID-19 pandemic itself may be a confounding factor, exacerbating the inability to afford care. Kindly clarify if this has been explored.
*Response: Our survey did ask whether being furloughed/unemployed was due to the pandemic. We now report that approximately 4% of this group reported that it was due to the pandemic (Results, lines 225-226). This is now mentioned again in the Discussion, lines 314-316.*

e. As stated in the limitations, the sample recruitment is primarily from ResearchMatch which may not be representative of general population. As per the ResearchMatch site, persons of specific race are overrepresented, as are some of the States in the US. This could possibly skew the data either way. This may kindly be clarified if there is any published data from general population where care avoidance was stratified according to race and demography, and how the care avoidance seen in this study group compares with the general population data.
*Response: In the Discussion (lines 327-329) and in the final paragraph of the Limitations (lines 410-413), we report that care avoidance in our study was significantly higher among younger and non-White participants, which is in line with findings from Czeisler et al (2020), who surveyed a nationally representative sample. Our study also found that those with poor mental health were more likely to avoid care, which supports findings of another study that used a nationally representative sample (Ganson et al 2020). This was noted in the Discussion (lines 332-334) and Limitations (lines 410-413).*

Others:
3. The proposed Figure 1 is an Onion diagram for groups of factors influencing care avoidance. However, the enlisted variables in the smaller circle are not a subset of those enlisted in the larger circle (sequentially). A Venn diagram or other similar options may offer better representation of the hypothesis used.
*Response: We appreciate this comment but would like to respectfully contend that our figure is a better illustration of our hypotheses compared to, for example, a Venn diagram or similar option. Our figure is based on a socioecological model of health which has been endorsed by the Centers for Disease Control and does, in fact, view the individual as the center (innermost circle). The individual’s behavior related to care seeking/care avoidance is influenced by their most immediate environment (second circle), which in our study included the family and media, as well as by the healthcare organization/institution itself (third circle). We now include additional text in the Introduction (lines 71-76) to strengthen our rationale for using this model, and we have added a new supporting reference (Kilanowski, 2017). Importantly, this model also guided our statistical analyses, with the regression analyses adding individual, environment, and organizational variables in separate steps. This has been reiterated in the data analysis section, lines 188-189.*

4. Line 13, please check if reference #11 is correct for the statement. It seems reference #12 might be more appropriate.
*Response: Our thanks to the reviewer for careful reading of our paper. We had cited reference #12 on line 13 (line 14 in the revised manuscript) when in fact the correct reference was #11. This has been corrected. Please note that the references have been re-numbered starting from reference #12 in the revised manuscript.*

**Reviewer #2 comments:**
This cross-sectional online survey by Bengt B. Arnetz et al., 2021 examined reasons for the avoidance of in-person care among COVID-19 patients who reported that they needed it in the United States of America from late October to early December 2020.

The study used a 45-item online survey instrument administered via Qualtrics using the following variables:

Dependent (outcome) variable: care avoidance
Independent variables: categorized as patient related; environment related; and institutionally related variables

Out of the total of 3840 persons who anonymously responded to the survey, a reasonably large sample size (n=3373) fulfilled the inclusion criteria and were analyzed using multivariable logistic regression. Consent was obtained from participants and ethical clearance granted by the Michigan State University Institutional Review Board.

The authors made all data underlying the findings fully available. The data was tested for representativeness, analyzed using descriptive and inferential statistics which were rigorous and appropriate.

Results obtained revealed that 257 (7.6%) respondents avoided in-person care during the early phase of the pandemic citing reasons:

Patient related:
young age: inability to afford care; greater COVID-19 related stress

Environment related:
infrequent discussions about COVID with family and friends

Institution related:
negative patient health care experience rating; poor awareness of the institution’s safety protocol; low ratings of the institution’s effectiveness in communicating their safety protocol

Overall, the study predicted 11.9% of the variance in 30 care avoidance.

Discussions of the results were robust, citing similar studies conducted during previous SARS-CoV-1, H1N1 and MERS pandemics. However, the study, in my view, excluded important variables from the regression model: needed urgent care, needed emergency care, chronic conditions, COVID-19 diagnosis, general health rating, consuming COVID-related content on news media. Although the authors did this “due to lack of independent relationship with care avoidance”, nevertheless this exclusion may be an important source of bias to the study.
*Response: We thank the reviewer for this important comment. As stated in the first paragraph of the Discussion (lines 288-293), this was an exploratory study. We had originally included variables related to the need for certain kinds of care as well as presence of chronic conditions but found that these were not independently related to care avoidance. Moreover, we were forced to exclude additional variables due to multicollinearity to establish a more parsimonious model. Nevertheless, we agree that exclusion of these variables could bias our results, and this is now mentioned in the Limitations section (lines 418-422).*

Conclusions are in line with the findings

Writing quality and clarity: Satisfactory
References: The manuscript employed the use of Harvard style referencing but requires editing to correct some errors noticed e.g., Listing of references: Shouldn’t this be in alphabetical order? Shouldn’t the journal name be italics? Shouldn’t the list of authors that are more than 5 be reflected as et al? e.g. reference number 7 could just be stated as Czeisler, M.E. et al. ( 2020).
I suggest the authors should revise Harvard referencing style and make necessary corrections.
*Response: PLoS One requires the Vancouver style for references, which is what we have utilized. However, several of our citations were entered incorrectly. We have carefully revised and corrected the entire reference list. Please note that several new references have been added (highlighted in yellow).*

**Reviewer #3 comments:**
The manuscript requires corrections throughout. The language needs improvement including sentence and paragraph structure in all areas of the manuscript. There is need for improvement to the referencing. Statements are made without references in several areas including the Discussion. The presentation of the results are very difficult to read and understand and requires significant improvement through revision. The Discussion is weak and the limitations are deficient.
*Response: We have carefully revised our paper and we have improved sentence and paragraph structuring throughout. We have also included additional references in the Discussion section. Both the Discussion and Limitation sections have been revised according to the comments provided by Reviewers 1 and 2.*

[Note: HTML markup is below. Please do not edit.]

Reviewers' comments:

Reviewer's Responses to Questions

**Comments to the Author**

1. Is the manuscript technically sound, and do the data support the conclusions?

The manuscript must describe a technically sound piece of scientific research with data that supports the conclusions. Experiments must have been conducted rigorously, with appropriate controls, replication, and sample sizes. The conclusions must be drawn appropriately based on the data presented.

Reviewer #1: Yes

Reviewer #2: Yes

Reviewer #3: Partly

2. Has the statistical analysis been performed appropriately and rigorously?

Reviewer #1: Yes

Reviewer #2: Yes

Reviewer #3: I Don't Know

3. Have the authors made all data underlying the findings in their manuscript fully available?

The [PLOS Data policy](https://urldefense.com/v3/__http:/www.plosone.org/static/policies.action*sharing__;Iw!!HXCxUKc!kIsn2J1i01BwNb1WuWY584TDx-7-rOz6f8CZdCouPb-AWbyvxnWajg0hdMA5IiA$) requires authors to make all data underlying the findings described in their manuscript fully available without restriction, with rare exception (please refer to the Data Availability Statement in the manuscript PDF file). The data should be provided as part of the manuscript or its supporting information, or deposited to a public repository. For example, in addition to summary statistics, the data points behind means, medians and variance measures should be available. If there are restrictions on publicly sharing data—e.g. participant privacy or use of data from a third party—those must be specified.

Reviewer #1: Yes

Reviewer #2: Yes

Reviewer #3: Yes

4. Is the manuscript presented in an intelligible fashion and written in standard English?

PLOS ONE does not copyedit accepted manuscripts, so the language in submitted articles must be clear, correct, and unambiguous. Any typographical or grammatical errors should be corrected at revision, so please note any specific errors here.

Reviewer #1: Yes

Reviewer #2: Yes

Reviewer #3: No

5. Review Comments to the Author

Please use the space provided to explain your answers to the questions above. You may also include additional comments for the author, including concerns about dual publication, research ethics, or publication ethics. (Please upload your review as an attachment if it exceeds 20,000 characters)

Reviewer #1: Overall an interesting piece of work that provides insights into patient perspective on in-person care avoidance during a pandemic. The results can potentially feed into care provider policies and improve services by addressing patient concerns and apprehensions during the current pandemic and beyond.
1. Methodology: It is not entirely clear whether telemedicine is being considered a sub-set of in-person care for the purpose of analysis in this study. Line # 337-338. For example, in primary care, a large majority of in-person care visits were transferred to telemedicine visits. Further, as in reference #12, (Alhadramy OM, Sr. The Structure and the Outcome of Telephone-Based Cardiac Consultations During Lockdown: A Lesson From COVID-19. Cureus. 2020), the conclusion states that: When standard face-to-face cardiac consultations are compromised, a structured telephone-based consultation is considered feasible, seems effective, and promising alternative method of delivering the utmost cardiac care to the community. It might be helpful if the authors mention that whether telemedicine was considered suitable alternative to physical in-person care, and how this has been dealt in the analysis.

2. Discussions:
a. It might be good to draw comparisons with results from other similar study at reference #7 (Czeisler ME, Marynak K, Clarke KEN, Salah Z, Shakya I, Thierry JM, et al. Delay or Avoidance of Medical Care Because of COVID-19-Related Concerns - United States, June 2020. MMWR Morb Mortal Wkly Rep. 2020). This would specifically be regarding
i. Is the current discussion on avoidance of in-person care, a subset of care avoidance discussed in reference #7?
ii. What is the possible influence of use of different tools for data collection on the results?
b. Line# 291-293: Our hypothesized model also included environment-related variables such as consuming COVID-related media news and the politics related to the pandemic, as we reasoned they might instill fear and panic, thereby influencing care avoidance. Although, as per the analysis, the variable did not have influence on patient decision in the analysis, this could have contributed to COVID related stress that had influence.
c. Kindly clarify if institutional factors like prioritization for COVID-19 patients and resource diversion could have been a factor for avoidance of in-person care?
d. Out of the seven key factors found to be associated with care avoidance, age, ability to afford care and HCO experience are not directly COVID-19 related. However, COVID-19 pandemic itself may be a confounding factor, exacerbating the inability to afford care. Kindly clarify if this has been explored.
e. As stated in the limitations, the sample recruitment is primarily from ResearchMatch which may not be representative of general population. As per the ResearchMatch site, persons of specific race are overrepresented, as are some of the States in the US. This could possibly skew the data either way. This may kindly be clarified if there is any published data from general population where care avoidance was stratified according to race and demography, and how the care avoidance seen in this study group compares with the general population data.
Others:
3. The proposed Figure 1 is an Onion diagram for groups of factors influencing care avoidance. However, the enlisted variables in the smaller circle are not a subset of those enlisted in the larger circle (sequentially). A Venn diagram or other similar options may offer better representation of the hypothesis used.
4. Line 13, please check if reference #11 is correct for the statement. It seems reference #12 might be more appropriate

Reviewer #2: This cross-sectional online survey by Bengt B. Arnetz et al., 2021 examined reasons for the avoidance of in-person care among COVID-19 patients who reported that they needed it in the United States of America from late October to early December 2020.

The study used a 45-item online survey instrument administered via Qualtrics using the following variables:

Dependent (outcome) variable: care avoidance
Independent variables: categorized as patient related; environment related; and institutionally related variables

Out of the total of 3840 persons who anonymously responded to the survey, a reasonably large sample size (n=3373) fulfilled the inclusion criteria and were analyzed using multivariable logistic regression. Consent was obtained from participants and ethical clearance granted by the Michigan State University Institutional Review Board.

The authors made all data underlying the findings fully available. The data was tested for representativeness, analyzed using descriptive and inferential statistics which were rigorous and appropriate.

Results obtained revealed that 257 (7.6%) respondents avoided in-person care during the early phase of the pandemic citing reasons:

Patient related:
young age: inability to afford care; greater COVID-19 related stress

Environment related:
infrequent discussions about COVID with family and friends

Institution related:
negative patient health care experience rating; poor awareness of the institution’s safety protocol; low ratings of the institution’s effectiveness in communicating their safety protocol

Overall, the study predicted 11.9% of the variance in 30 care avoidance.

Discussions of the results were robust, citing similar studies conducted during previous SARS-CoV-1, H1N1 and MERS pandemics. However, the study, in my view, excluded important variables from the regression model: needed urgent care, needed emergency care, chronic conditions, COVID-19 diagnosis, general health rating, consuming COVID-related content on news media. Although the authors did this “due to lack of independent relationship with care avoidance”, nevertheless this exclusion may be an important source of bias to the study.

Conclusions are in line with the findings

Writing quality and clarity: Satisfactory
References: The manuscript employed the use of Harvard style referencing but requires editing to correct some errors noticed e.g., Listing of references: Shouldn’t this be in alphabetical order? Shouldn’t the journal name be italics? Shouldn’t the list of authors that are more than 5 be reflected as et al? e.g. reference number 7 could just be stated as Czeisler, M.E. et al. ( 2020).
I suggest the authors should revise Harvard referencing style and make necessary corrections.

Reviewer #3: The manuscript requires corrections throughout. The language needs improvement including sentence and paragraph structure in all areas of the manuscript. There is need for improvement to the referencing. Statements are made without references in several areas including the Discussion. The presentation of the results are very difficult to read and understand and requires significant improvement through revision. The Discussion is weak and the limitations are deficient.

6. PLOS authors have the option to publish the peer review history of their article ([what does this mean?](https://urldefense.com/v3/__https:/journals.plos.org/plosone/s/editorial-and-peer-review-process*loc-peer-review-history__;Iw!!HXCxUKc!kIsn2J1i01BwNb1WuWY584TDx-7-rOz6f8CZdCouPb-AWbyvxnWajg0hKEGjbd8$)). If published, this will include your full peer review and any attached files.


If you choose “no”, your identity will remain anonymous but your review may still be made public.


**Do you want your identity to be public for this peer review?** For information about this choice, including consent withdrawal, please see our [Privacy Policy](https://urldefense.com/v3/__https:/www.plos.org/privacy-policy__;!!HXCxUKc!kIsn2J1i01BwNb1WuWY584TDx-7-rOz6f8CZdCouPb-AWbyvxnWajg0h031ANsw$).

Reviewer #1: **Yes:** Vineet Bhatia

Reviewer #2: **Yes:** Haruna Ismaila Adamu, MBBS; MPH; PhD

Reviewer #3: **Yes:** Glennis Andall-Brereton

[NOTE: If reviewer comments were submitted as an attachment file, they will be attached to this email and accessible via the submission site. Please log into your account, locate the manuscript record, and check for the action link "View Attachments". If this link does not appear, there are no attachment files.]

While revising your submission, please upload your figure files to the Preflight Analysis and Conversion Engine (PACE) digital diagnostic tool, [https://pacev2.apexcovantage.com/](https://urldefense.com/v3/__https:/pacev2.apexcovantage.com/__;!!HXCxUKc!kIsn2J1i01BwNb1WuWY584TDx-7-rOz6f8CZdCouPb-AWbyvxnWajg0hP08l1XU$). PACE helps ensure that figures meet PLOS requirements. To use PACE, you must first register as a user. Registration is free. Then, login and navigate to the UPLOAD tab, where you will find detailed instructions on how to use the tool. If you encounter any issues or have any questions when using PACE, please email PLOS at [figures@plos.org](mailto:figures@plos.org). Please note that Supporting Information files do not need this step.
