# Supplementary material for: Patient-reported factors associated with avoidance of in-person care during the COVID-19 pandemic: Results from a national survey
Source: PLoS One. 2022 Aug 5;17(8):e0272609. doi: 10.1371/journal.pone.0272609 (PMC9355185; doi:10.1371/journal.pone.0272609)
Supplement: Attachment — Submitted filename: Response to reviewer comments.docx [file pone.0272609.s003.docx]

**Manuscript PONE-D-21-35375R1: Response to reviewer comments**

Dear Editor:

Our responses to the reviewer comments are in italics below each comment. Our thanks to the reviewers for their comments that have helped us to improve our paper.

Reviewer #1: Thanks for addressing most of the comments and providing justification response to others. Best wishes!

*Response: Thank you!*

Reviewer #2: This cross-sectional online survey was conducted in the United States by Arnetz, B. B., et al., 2022. from late October to early December 2020 with a questionnaire administered using ResearchMatch and Facebook. The study explored why patients avoided needed in-person care during the early phase of the COVID-19 pandemic in the United States from three perspectives: patient, external environment, and institution.

Dependent (outcome) variable: care avoidance
Independent variables: categorized as patient related; environment related; and institutionally related Variables

Out of the total of 3840 persons who anonymously responded to the survey, a reasonably large sample size (n=3373) fulfilled the inclusion criteria and were analyzed using multivariable logistic regression. Consent was obtained from participants and ethical clearance granted by the Michigan State University Institutional Review Board.

Statistical analysis was conducted using IBM SPSS Statistics version 26. A two-sided P value <0.05 represented statistical significance. A principal component analysis of the three safety rating variables gave a single factor solution with an alpha of .88, thus justifying the combination of the variables into an index

Results revealed that a total of 3840 patients completed the survey. Patients who reported that they had not needed care (n=423, 11.0%) and those not responding to the question (n=45, 1.2%) were excluded from this analysis, leaving a sample size of 3372. Out of a total of 3372 respondents who reported that they needed in-person care during the early phase of the pandemic, 257 (7.6%) avoided it. Patient-related variables associated with avoiding needed care included younger age (odds ratio (OR), 1.46, 95% CI 1.11 to 1.94, p<0.01; 53 <45 y/o vs 45+), inability to afford care (OR=1.65, 95% CI 1.17 to 2.34, p<0.01), and greater COVID-related stress (OR=1.36, CI 1.01 54 to 1.83, p<0.05). More frequent discussions about COVID with family and friends was the only significant environment-related avoidance of care variable (OR=1.39, 95% CI 1.01-1.91, p<.05). Institution-related care avoidance variables 57 included a negative patient healthcare experience rating (OR 1.83, 95% CI 1.38 to 2.42, 58 p<0.001), poor awareness of the institution’s safety protocol (OR=1.79, 95% CI 1.28 to 2.51, p<0.01), and low ratings of the institution’s effectiveness in communicating their safety protocol 60 (OR=3.45, 95% CI 1.94 to 6.12, p<0.001). The final model predicted 11.9% of the variance in care avoidance.

The authors made all data underlying the findings fully available. The data was tested for representativeness, analyzed using descriptive and inferential statistics which were rigorous and appropriate.

Discussions of the results were robust, citing similar studies conducted both within and outside Nepal.

Conclusions are in line with the findings

Writing quality and clarity: Satisfactory
Other observations:
1. Limitations of the study: The authors did well to mention the limitations of the study, including how these limitations should be addressed by future studies going forward
2. Inclusion/exclusion criteria clearly explained
References: The manuscript employed the use of Harvard style referencing but requires editing to correct some errors noticed e.g., Listing of references: Shouldn’t this be in alphabetical order? Shouldn’t the journal name be italics? Shouldn’t the list of authors that are more than 5 be reflected as et al?
I suggest the authors should revise the reference per the Vancouver referencing style and make necessary corrections.

*Response: Per the journal submission guidelines (https://journals.plos.org/plosone/s/submission-guidelines), PLOS ONE requires that references follow the Vancouver style, which we have utilized in our paper. However, based on comments by reviewer #4, our entire reference list has been updated to the most recent Vancouver style. According to guidelines, references are in the order in which they are cited, not in alphabetical order. Journal names are based on the NCBI database abbreviations and are not italicized.*

Reviewer #4:
I thank the authors to address all the reviewer's comments. However, two minor comments are still remaining:
1. The reference style must be updated per the Vancouver style.

*Response: Please see our response to the previous point (Reviewer #2).*

2. The following paper discuss on the Covid-19 impacts on the cancer care.
- <https://www.ncbi.nlm.nih.gov/pmc/articles/PMC8184167/>
It is suggested authors cite it and make a summarize this and similar articles regarding the Covid-19 impacts on patients cares in different disease entities (in 1-2 paragraphs in the Introduction).
*Response: Our thanks to the reviewer for this interesting paper and for this suggestion. We have added a paragraph in the Introduction, lines 40-54, where we review studies of the impact of the pandemic on specific disease conditions.*

While revising your submission, please upload your figure files to the Preflight Analysis and Conversion Engine (PACE) digital diagnostic tool, [https://pacev2.apexcovantage.com/](https://urldefense.com/v3/__https:/pacev2.apexcovantage.com/__;!!HXCxUKc!w2pdEhLwyO7XGNgpoPjqwLAjT_ZM-s3GVTDXni4Ta6yRbB7rPQEE_kLp_uSv-6GyGC_v0lJerFUH56aT$). PACE helps ensure that figures meet PLOS requirements. To use PACE, you must first register as a user. Registration is free. Then, login and navigate to the UPLOAD tab, where you will find detailed instructions on how to use the tool. If you encounter any issues or have any questions when using PACE, please email PLOS at [figures@plos.org](mailto:figures@plos.org). Please note that Supporting Information files do not need this step.

*Response: We have revised our Figure using the PACE digital diagnostic tool.*
